# A qualitative study of evidence-based therapeutic process in mental health services in Ghana– context-mechanisms-outcomes

**DOI:** 10.1186/s12913-021-06993-1

**Published:** 2021-09-25

**Authors:** Eric Badu, Anthony Paul O’Brien, Rebecca Mitchell, Akwasi Osei

**Affiliations:** 1 School of Nursing and Midwifery, Faculty Health and Medicine, The University of Newcastle, Callaghan, Australia; 2 Faculty of Health, Southern Cross University, Lismore, Australia; 3 Macquarie Business School, Macquarie University, Sydney, Australia; 4 Ghana Mental Health Authority, Ghana Health Services, Accra, Ghana

**Keywords:** Therapeutic relationship, Technical competencies, Mental health professionals, Quality, Mental health services, Ghana

## Abstract

**Background:**

Evidence-based clinical practice is an inherent component of mental health professional practice in developed countries. However, little is known about professional perspectives of evidence-based practice in mental in developing countries such as Ghana. This paper describes the processes involved in the delivery of best practice in Ghana. The paper reports on a realistic evaluation of mental health nurses and allied health professionals’ views on the evidence-based therapeutic process in Ghana.

**Methods:**

A purposive sample of 30 mental health professionals (MHPs) was recruited to participate in semi-structured, in-depth interviews. Thematic analysis was used to analyse the data. A program theory of Context + Mechanism = Outcome (CMO) configuration was developed from the analysis.

**Results:**

The thematic analysis identified two contexts, mechanism and outcome configurations (themes): 1) technical competency stimulates evidence-based mental health services, and 2) therapeutic relationship building ensures effective interaction. The study demonstrates that contextual factors (technical competencies and therapeutic relationship building) together with mechanisms (intentional and unintentional) help to promote quality in mental health service provision. However, contextual factors such as a lack of sign language interpreters yielded unintended outcomes including barriers to communication with providers for consumers with hearing impairment and those from linguistic minority backgrounds.

**Conclusion:**

Government stakeholders and policymakers should prioritise policies, periodic monitoring and adequate financial incentives to support the mechanisms that promote technical competence in MHPs and the building of therapeutic relationship.

**Supplementary Information:**

The online version contains supplementary material available at 10.1186/s12913-021-06993-1.

## Background

Evidence-based practice (EBP) is a widespread expectation in the delivery of mental health services [1, 2]. The process informed by EBP is fundamental to psychological growth and relevant change in the lives of consumers. Several theoretical and conceptual frameworks, as well as principles, have been proposed to govern the implementation of EBP, which encapsulates person-centred, strength-based and people-first service provision, and the active involvement of consumers in decisions about treatment and recovery [1, 2]. The process incorporates individual clinical expertise with the best available evidence from systematic reviews, randomised clinical trials, quasi-experimental, pre-post and correlational designs, as well as continuous clinical education and upskilling [1, 2].

Scholars have proposed several initiatives that could enhance the evidence-based process in mental health services [1, 2]. For example, Anthony and Mizock [1] recommended that the process of EBP should encompass a positive therapeutic relationship, goal-setting, expectations and hope, consumers being taught new skills, self-awareness, as well as consumers feeling supported. The therapeutic relationship, which constitutes a key component of EBP, is a multi-faceted concept used to describe a collaborative and transformative relationship formed between Mental Health Professionals (MHPs) and consumers and their family caregivers [1, 3, 4]. Specifically, Kornhaber, Walsh [5] described this relationship as caring, supportive and non-judgemental behaviour, embedded in the process of delivering services. Achieving this relationship is underpinned by several ethical principles, including mutual trust, respect and dignity, as well as the nurturing of hope [6, 7].

Several studies have suggested interrelated attributes for characterising this therapeutic relationship [5, 8–10]. The therapeutic relationship has largely been characterised as building a rapport [8, 9], therapeutic listening and responding to consumers’ emotions [5]. The relationships are mostly categorised into individual (consumer, family caregivers and mental health professionals) and organisational attributes (environmental attributes) [3, 11]. The individual attributes align with factors in consumer and family caregivers as well as MHP factors that enable or hinder the establishment of the therapeutic relationship. This might include consumers’ insights, knowledge, humour and communication challenges (verbal and non-verbal), and work fatigue, skills, competency and attitudes of MHPs [3, 11, 12]. Conversely, the organisational attributes constitute factors influenced by the treatment setting or environment. These attributes may include the philosophy of care (e.g. paternalistic and medical model traditions, organisational policies and practices, increasing workload, staff shortages and a large number of patients [3, 11, 13, 14].

Research has highlighted that the therapeutic relationship could have a positive effect on the quality of mental health services [1, 8, 15]. For example, for consumers, the therapeutic relationship promotes personal recovery, increased satisfaction with services, quality of life, reduced symptoms and functionality [4, 15, 16]. Given these significances, mental health stakeholders are increasingly advocating for an evidence-based process to enhance quality mental health services. Although some studies have suggested the need to understand the evidence-based process in mental health nursing, there is little evidence to support the development of effective therapeutic relationship building, particularly in clinical practice. Research on therapeutic relationship building has been limited to general health services, particularly in developed countries, eg. Australia [8, 12] UK [17] USA, and Germany) and Middle Eastern countries [3, 18], eg. Iran and Saudi Arabia).

In developing countries including Ghana, there is limited evidence on issues related to EBP, which is integral to the recovery of consumers. Although there has been increasing efforts to improve the living conditions of consumers with mental illness and, thus has resulted in increasing empirical studies on mental health services, to date, the evidence has focused on weaknesses in the health system [19], challenges in policy implementation, enablers and barriers in accessing services [20], and treatment pathways using conventional middle-range theories [21, 22]. None of these studies has attempted to understand the perspectives of MHPs regarding the evidence-based therapeutic process. To address this gap, a qualitative study, using realistic evaluation as part of a concurrent mixed-methods approach, was undertaken to explore mental health professional’s views on the evidence-based therapeutic process in psychiatric facilities in Ghana.

### Middle-range theories supporting evidence-based therapeutic processes

Several theories have been proposed to inform EBP in health service delivery, which applies to mental health. Examples of these middle-range theories include the Donabedian theory of quality of care [16] and Hildegard Peplau’s middle-range theory of interpersonal relationships [23–25].

The process component of the Donabedian middle-range theory describes EBP as the actual treatment stage and is comprised of consumer-based interpersonal relationships and the technical skills of the mental health professionals. According to the Donabedian theory, consumer-based interpersonal relationships highlight the therapeutic relationship between consumers and providers. The technical skills describe clinicians’ knowledge about appropriate interventions and best practices, as well as their ability to accurately assess consumer problems [16].

Hildegard Peplau’s middle-range theory states that consumer-based interpersonal relationships must go through three phases, including orientation, working (identification and exploitation) and termination (resolution) [23, 25]. The therapeutic interaction between consumers and service providers is central in each of the three phases. For example, at the orientation phase, providers meet consumers and gain essential information. Service providers make assessments about consumers through a collaborative and interdisciplinary plan of care at the working phase, to determine the best evidence-based interventions. Finally, service providers create discharge plans, which include symptom management and recovery planning at the termination phase [24, 25].

## Methods

### Study setting

The study was conducted between June 2019 and November 2019 at three psychiatric facilities in Ghana. Two of the facilities are specialised psychiatric hospitals, while the third was a unit within a general hospital. The two psychiatric facilities are located in the Greater Accra region and the psychiatric unit is located at the Komfo Anokye Teaching Hospital in the Ashanti region. All three facilities operated based on a shared goal and focus of treatment.

### Research design and approach (phase 1 – initial theory and assumption)

This paper focuses on the qualitative component of a larger, concurrent mixed-method study design, which draws on the principles of realist evaluation to explore mental health professionals’ perspectives on evidence-based processes in the provision of mental health services. This qualitative component explores the subjective views of mental health professionals in the process of delivering mental health services. In particular, the qualitative data was used as secondary to the quantitative objective measure of the outcome of mental health services. It enabled the researchers to interact with mental health professionals and to listen to their subjective experiences in the process of providing services to consumers. It also helps to generate a rich and in-depth exploration of the therapeutic process in mental health service delivery [26]. The naturalistic, holistic view of qualitative methods is relevant to better understand the therapeutic process, rather than simply the quantitative measure of outcome. Specifically, the realistic evaluation cycle was conceptualised into phases and started with initial theory development [27, 28]. Data were captured through semi-structured interviews, surveys, and a review of the literature. The literature review [19, 21], concepts [16, 26] and quantitative components [29, 30] informing this realistic evaluation have previously been published. The realistic evaluation provides a unique perspective on services and is grounded in theory [27, 31]. The central tenet of this realist methodology is that the services may work differently in different contexts [28, 32].

This study offers ways to address what works (or not), when, why, for whom and under what circumstances to promote evidence-based therapeutic interaction in service provision [33, 34]. The therapeutic processes in mental health services have traditionally been tested and evaluated through middle-range theory (e.g. Donabedian theory or Hildegard Peplau’s theory). However, such theories do not offer ways to identify contextual factors, nor mechanisms that could promote the therapeutic interaction between consumers and providers. This realistic evaluation involves the development and refinement of a program theory, which is explained according to Pawson and Tilley’s (1997) formula: Context + Mechanism = Outcome.

The Donabedian middle-range theory and Hildegard Peplau’s theory on interpersonal relations were used to explain the therapeutic processes of providing mental health services [35].. The qualitative data were empirically tested to develop a final program theory that explains contextual factors and mechanisms that enhance the evidence-based therapeutic processes in delivering mental health services (Tables 2 and 3). The experiences of MHPs based on their therapeutic interactions with consumers were used to refine the program theory. The next step involved developing the underlying assumptions, to articulate the program theory [27, 31, 36].

### Data collection (phase 2 – recruitment and fieldwork)

Qualitative methods including field notes and in-depth interviews were used to collect data from MHPs. The clinical coordinators in each psychiatric facility facilitated the recruitment of participants. The researchers reviewed the list of MHPs in each of the selected facilities and selected those who met the eligibility criteria. MHPs were included if they had worked for at least three years in the respective psychiatric facility and provided daily routine mental health services. Based on the inclusion criteria, we invited 38 MHPs through email, face-to-face or telephone call. The invitation contained a letter of introduction, participant information sheets, and consent forms. Similarly, the head of each psychiatric facility sent a memo detailing the research and advertised it on the notice boards in each department. A total of 30 MHPs agreed and were purposively recruited to participate in an in-depth interview. The data were collected until saturation, when no new information emerged.

The interviews were conducted using a structured interview guide (Additional file 1) and captured information on contextual factors and mechanisms that could improve therapeutic processes in mental health services. The interview guide was developed using relevant information according to the Donabedian middle-range theory [16] and Papau theory on interpersonal relations. Specifically, the content of the interview guide covered questions on the evidence-based therapeutic process in providing mental health services. For example, the questions focused on the technical skills of providers, the training and professional development plan and consumer-based interpersonal relationships [23–25]. In particular, the questions also covered clinicians’ knowledge of appropriate interventions and best practices, as well as their ability to accurately assess the problems of consumers [16].

In the interview sessions, MHPs were briefed about the research objectives, procedures and the consent process. The right of participants to safeguard their anonymity and integrity was respected. Each MHP was informed of the study’s aims, methods, and consent to participation, potential risk/benefits and privacy/confidentiality. The MHPs signed an informed consent form prior to participation. The lead author conducted the interviews and therefore read the questions and recorded (with permission) responses, using an audio recorder. The interviewer has several years of experience in conducting qualitative interviews, particularly in the health facility setting. Although the interviewer understands the mental health systems, thus based on previous research, he did not have any work, social and emotional links with the participants. The interviewer also monitored all the interview process, including gestures and memos. We assigned unique identifiers to the audio recordings, to maintain the confidentiality of the participants. All interviews were conducted in English, which is the primary language of conversation in formal settings in Ghana. The interviews were conducted at the psychiatric facilities, in the clinical staff common rooms, privately and separately; that is, no interview was witnessed by a clinical coordinator or service provider. Each interview took approximately 45 to 60 min.

### Data analysis (phase 3 – analysis and configuration of CMO)

The data from field notes and in-depth interviews were analysed using thematic analysis, conducted according to Braun and Clarke [37] approach to categorising and connecting data, to develop a realist theory (Tables 2 and 3). This process involved transcribing, reading, familiarising with the data, generating initial codes, searching for themes, reviewing themes, and rigorous interpretation of data.

An independent transcriber transcribed the 30 de-identified audio recordings into an MS Word document. The interviewer listened to the audio recordings and reviewed the transcripts and field notes. The transcribed data was then entered into NVivo 12 for analysis. The lead author, working closely with all co-authors, performed the coding process. As recommended by Saldaña [38], initial coding were conducted to develop inductive codes. The authors revised the initial codes to better understand which ones to include in new inductive codes. The authors then performed focused coding, thus successive coding, of all the remaining data to identify additional codes. In the focused coding, the most significant initial codes were used as provisional categories for checking across all the transcribed data. The focused coding continued until saturation was reached – no new ideas emerged from successive coding.

The analysis identified a total of 49 inductive codes. The codes were categorised into two major themes that explain the evidence-based therapeutic process in mental health service delivery (Table 2). For example, theme 1 *“technical competency stimulates evidence-based mental health services”* consist of 19 inductive codes, whilst theme 2 *“therapeutic alliance-building ensure effective interaction*” consist of 29 inductive codes (Tables 2 and 3).

Memos were written throughout the coding process to record emerging conceptual links and observations from the data. The identified codes were then used to develop the context, mechanism and outcome (CMO domain) categories. The context describes the policy background and mental health systems as well as the organisational setting where the services are being delivered [27, 39]. Also, the mechanism describes what makes the services work. They refer to the intentional and unintentional approaches and strategies that are implemented to improve the therapeutic process in mental health service delivery [27, 39]. The outcome in our realistic evaluation refers to the intended or unintended changes that are achieved in the lives of consumers as a result of the efforts by the therapeutic process [27, 39]. The first CMO configuration (theme one) and second CMO (theme two) consist of all codes that explain contextual factors, and mechanisms that could enhance the outcome of mental health services (Tables 2 and 3). A visual model was developed to establish the patterns of CMO across codes and cases. As illustrated in Table 2, the patterns denoted the causal pathways, and thus the contextual elements that could trigger or influence mechanisms, to produce mental health service outcomes. The most prominent quotes and words from MHPs that were relevant to each of the context, mechanism and outcome are used to support the findings.

## Results

### Background information

Table 1 provides demographic data from MHPs. The average age was 36.4 years and more than half of MHPs (17/30; 56.67%) were female. Most MHPs (20/30; 66.67%) were married, while 30% were single. A third of the MHPs were registered mental health nurses, while 23.33% were psychiatrists. The highest level of education was a postgraduate degree (e.g. MPH, MSc, MPhil and MBChB), and the average professional experience was 36 years.

**Table 1:** Background information of MHPs

ID	Health Facility	Profession	Qualification	Age	Gender	Marital status	Experience
001	Facility 1	Mental health nurse	Diploma in Nursing	52	Male	Married	24
002	Facility 3	Occupational Therapist	BSc Occupational Therapist	26	Male	Single	3
003	Facility 3	Mental Health Nurse	BSc Mental Health Nursing	34	Female	Married	11
004	Facility 3	Clinical Psychologist	MPhil Clinical Psychologist	35	Male	Married	8
005	Facility 3	Psychiatrist	MBChB. MWACP	29	Male	Single	3
006	Facility 3	Psychiatrist	MBChB. MWACP	33	Male	Married	5
007	Facility 3	Psychiatrist	MBChB. MWACP	33	Male	Single	4
008	Facility 3	Lay Art Therapist	MPhil Art Education	29	Female	Married	3
009	Facility 3	Clinical Psychologist	MPhil Clinical Psychologist	53	Male	Married	10
010	Facility 2	Occupational Therapist	BSc Occupational Therapist	28	Male	Single	3
012	Facility 2	Community Mental Health Nurse	Diploma in CMHN	29	Female	Single	7
011	Facility 2	Occupational Therapist	BSc Occupational Therapist	25	Female	Single	3
013	Facility 2	Registered Mental Health Nurse	Diploma in Nursing	35	Female	Married	12
014	Facility 2	Community Mental Health Nurse	BSc Community Psychiatric Nurse	36	Female	Married	11
015	Facility 2	Community Mental Health Officer	Diploma in CMHO	34	Male	Married	9
016	Facility 2	Registered Mental Health Nurse	Bed. Health Sciences	35	Female	Married	18
017	Facility 2	Registered Mental Health Nurse	BSc Nursing	35	Female	Married	9
018	Facility 2	Clinical Psychologist	MPhil Psychology	50	Female	Married	3
019	Facility 2	Physician Assistant (Psychiatrist)	BSc Clinical Psychiatry	56	Male	Married	11
020	Facility 2	Registered Mental Health Nurse	BSc Mental Health Nursing	37	Female	Separated	8
021	Facility 2	Registered Mental Health Nurse	Bed. Health Science	35	Male	Married	10
022	Facility 1	Registered Mental Health Nurse	BSc General Nursing	41	Female	Married	12
023	Facility 1	Psychiatrist	MBChB. MWACP	35	Female	Married	6
024	Facility 1	Social Worker	BA, Post-graduate, MSc Development Economics	40	Male	Married	12
025	Facility 1	Registered Mental Health Nurse	Dip. RMN, BSc Nursing	35	Female	Single	11
026	Facility 1	Registered Mental Health Nurse	MPH Public, BSc Psychology, Diploma Nursing	39	Female	Married	16
027	Facility 1	Clinical Psychologist	MPhil Psychology	30	Female	Married	3
028	Facility 1	Resident Trained Psychiatrist	MBChB	29	Female	Single	4
029	Facility 1	Resident in Psychiatrist	MBChB	36	Female	Single	3
030	Facility 1	Resident	MBChB, MPH	48	Male	Married	12

*Min/Max; Mean (25/53; 36.4)*

### CMO configurations from the analysis

The thematic analysis identified two contexts, mechanism and outcome configurations (themes) as per the program theory (Tables 2 and 3). The findings are presented according to these contexts, mechanism and outcome, and are supported with relevant verbatim quotes from the transcribed data.

**Table 2:** CMO from the configuration

CMO from the configuration	Context + Mechanism = Outcome
CMO configuration one (theme one): Technical competency stimulates evidence-based mental health services	MHPs have received specialist training, practical skills, certified license as well as being regulated, monitored and supervised (**Context**). This is achieved through continuous learning, in-service training, professional development plan, multi-disciplinary team, clinical and academic training sessions (tutorials, presentations and discussions) (**Mechanisms**). These are helping to deliver quality mental health services, by reducing hospitalization days for consumers, improved MHPs knowledge and skills on current treatment methods, providing effective and efficient services to consumers, consumers to built confidence and trust in the care (**Outcome**) This notwithstanding, MHPs needed in-service training (aggression and conflict management, training in using psychological tools and test, customer care, health promotion, communication skills, infection prevention, operating ECT as well as dealing with difficult family caregivers), inadequate MHPs in clinical and allied health specialties (child and adolescent, forensics, geriatric psychiatry, addiction substance as well as social work and psychological services), adhering to western protocols and clinical judgement with on-going deliberation regarding the need to get a local protocol (**Mechanisms**)
CMO configuration two (theme two): Therapeutic relationship building ensure effective interaction	MHPs build the therapeutic relationship in psychoeducation, consultation, diagnosis, as well as family therapy session (**Context**). This was built through consumers involved in the care plan, considering consumers educational background, philosophical approach to admission (eg. voluntarily or involuntarily) dignity and respect, making consumers aware of the choices, building rapport, ethical principles (eg. respecting consumers privacy/confidentiality, dignity, preferences, informed consent, comfort, religion, gender, age, belief systems), need to stop wearing providers uniforms (**Mechanisms**) and this help equip consumers in management of the condition, enhance consumers’ knowledge and understanding, successful compliance with medications, consumers participating actively in the care plan (if educated), consumers feeling accepted, having hope and expectations, adhere to medications, making the service participatory, MHPs avoid being physically attacked or injured by angered consumers, promoting the quality of mental health services, help to avoid distinguishing consumers and providers on duty. (**Outcome**). This notwithstanding, MHPs have limited knowledge in sign language/no designated sign language interpreter to support communication, and using family caregivers, teachers or instructors or health professionals from other units to provide sign language support (**Mechanisms)** professional-consumers communication barriers (**Outcome**)

**Table 3:**
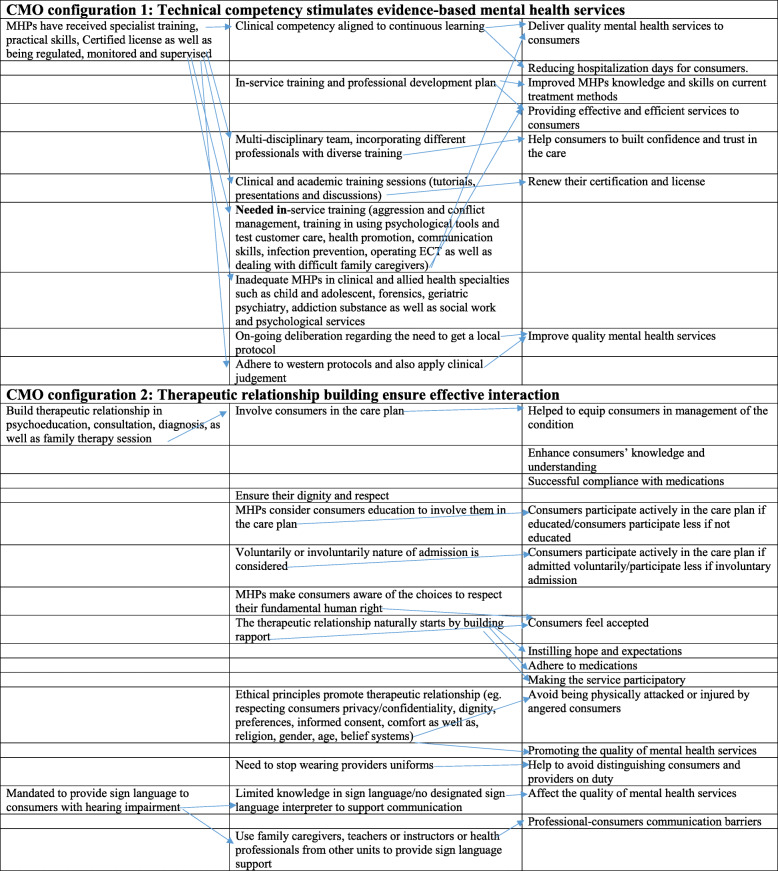
CMO from the configuration

### CMO configuration 1: technical competency stimulates evidence-based therapeutic process in mental health services

This context, mechanism and outcome configuration describe the effects of technical competency on the evidenced-based therapeutic process in mental health services. This context, mechanism and outcome was configured using 19 inductive codes emerging from the analysis. Most MHPs reported that they had received specialist training, practical skills, certified licensing as well as being regulated, monitored and supervised. The MHPs perceived that they had insights on the theories, concepts and practical skills in providing mental health services. Most participants expressed that their clinical competencies were achieved through mechanisms such as continuous learning, in-service training, professional development, multidisciplinary team involvement and clinical and academic training sessions (tutorials, presentations and discussions). Most participants confirmed that they received in-service training opportunities every quarter (e.g. at least 2 to 4 times a year). Some participants mentioned that as part of the professional development plan, the psychiatric facilities annually requested the training needs of every staff member. Several clinical and academic training sessions (tutorials, presentations and discussions) were organised in the psychiatric facilities for specific days to update MHPs on specific issues.

The participants explained:

Yes, almost every morning here we have our morning tutorials or meetings where we have discussions on various topics and we more or less teach each other, we have CPDs where we have lecturers from this country and sometimes from outside the country also coming to update us on relevant information so we always make sure that we update ourselves with relevant information and current information (Participant 7; psychiatrist; Facility 3).

Three MHPs (occupational therapist, psychiatrist and clinical psychologist) elucidated that the various professional associations for prescribers and allied health services organised training and academic sessions for MHPs to renew their certification and licence:

As part of our allied health association every year you have to go for professional development programs and accumulate a minimum of 12 points to be able to renew your registration with the council; yes so for that one throughout the year we go for continuous professional development programs to add on to our knowledge’ (Participant 10; Occupational therapist; Facility 2).

The contextual factors related to technical competencies and the unintended and intended mechanisms adopted by the psychiatric facilities influenced the outcomes of mental health services. For example, some MHPs reported that clinical competence, in-service training and professional experience mostly helped them to deliver quality mental health services to consumers. Three RMHNs also mentioned that their clinical competence and working within the multidisciplinary team helped consumers to build confidence and trust in the care provided, which in turn led to reducing in-patient bed days:

If the nurse is knowledgeable in how to ensure that the client gets well, he/she puts the knowledge into practice so that eventually the client doesn’t spend so much time on the ward because spending so much time on the ward means you are paying more, so that if there is the need for a progress report and maybe talking to a prescriber to ensure that the right thing is done it helps the nurse and it also helps the client in the long run. (Participant 21; RMHN; Facility 2).

If you can communicate well with your clients and your clients have confidence in you it will help them to come back for treatment whenever the need arises and it will also help the client to trust you because they know that you know what you are about. (Participant 16; RMHN; Facility 2).

Further, some MHPs (e.g. clinical psychologist, occupational therapist and RMHN) related that the in-service training had improved their knowledge and skills on current treatment methods, and subsequently enabled them to provide effective and efficient services to consumers:

It informs us of current trends in treatment, it informs in current trends in diagnosis and identifying psychopathologies and then also basically making our treatments as straightforward and as precise as possible. (Participant 4; Clinical psychologist; Facility 3).

Although participants believed themselves to have adequate clinical competence, they suggested several training needs that could further equip them with skills and practice to enable the provision of quality mental health services. For example, most participants suggested that they needed training in aggression and conflict management, in using psychological tools and tests, infection prevention, operating ECT, dealing with difficult family caregivers and health promotion, as well as customer care and communication skills:

We interact with these patients and we get patients becoming aggressive, so training in aggression, conflict management, training in the various therapies that we have and the current therapist that are being used elsewhere … we also need training in customer care, communication skills … training in even conflict management among staff, not just about the patient we care for but among the staff. (Participant 26; RMHN; Facility 1).

Moreover, participants described some challenges that could compromise their competence and the use of evidence-based treatment, including inadequate clinical and allied health specialties in child and adolescent mental health, forensics, geriatric psychiatry, addiction, substance abuse, social work and psychological services:

It would be good for us to have specific training in the different branches of psychiatry, further training in the different branches of psychiatry like forensic psychiatry, geriatric psychiatry, child and adolescent, addiction because we are more or less general psychiatrists so it would be good if we got like further training in these areas to make us even more efficient. (Participant 7; psychiatrist; Facility 3).

Yes, for the subspecialties, child, we need psychiatrists, we need forensics, we need substance addition subspecialists so all those things we are specialists providing the care but it could be better if we had people who have fellowship training such specialty skills but that’s our vision that in the next 5 years we are hoping to develop all those subspecialty trainings to get some of us to train in those fields as well. (Participant 6; psychiatrist; Facility 3).

… training is in general adult psychiatry so when it comes to such specialty services like child and adolescent forensics we have the general training. Our best-trained subspecialists are myself and my colleague who recently joined me. I have a masters in child and adolescent mental health which is not a clinical fellowship but at least some focused training in child and adolescent mental health and that’s about the best we have, everybody else here has general adult psychiatry so the geriatric service we are rendering, the forensic course reports are all based on general adult psychiatry knowledge that we have so in that sense we are probably not as resourced-rich as western countries. (Participant 6; Psychiatrist; Facility 6).

Some MHPs also added that while there was no local protocol to inform EBP, they adhered to international standards in the clinical practice. Some MHPs further recommended that there is an ongoing deliberation regarding the need to get a local protocol:

We adapt international standards slightly but we most of the time comply with international standards and if we have to make a few adjustments concerning local protocols cost usually has a role to play but most of the time international standards evidence-based medicine that is what we roll with and we keep modifying our treatment protocols as and when we need to. (Participant 7; Psychiatrist; Facility 3).

### CMO configuration 2: therapeutic relationship building ensures effective interaction

This context, mechanism and outcome configuration describes the influence of therapeutic relationship building on effective interaction between consumers and MHPs. This context, mechanism and outcome was configured using 29 inductive codes emerging from the analysis. As per the context of the program theory, most participants stated that they built therapeutic relationships through psychoeducation, consultation, diagnosis and family therapy sessions. The MHPs recounted that they involved consumers in the care plan, built rapport with them and ensured their dignity and respect. The analysis revealed that the involvement of consumers in the care plan was dependant on their educational background and the nature of admission (e.g. voluntarily or involuntarily). The MHPs believed that consumers who were educated or admitted voluntarily mostly knew about the condition and treatment options, and therefore, participated actively in the care plan:

… patients especially the more educated patients like to have interaction, like to be involved in the decision making on the treatment … by all means go online to be involved in the decision with the side effects profile, we weigh the options. (Participant 30; psychiatrist; Facility 1).

We don’t only involve patient or clients but we involve clients and their relatives to understand the condition the patient is going through … before the admission, we make this known to them … while the patient is going through the treatment, we make the relatives understand what the patient is going through so that even the management of the patient in our facility, they should also be able to better manage the patient after discharge. (Participant 24; Social worker; Facility 1)

… with the voluntary, because they have insight, they admit that they are mentally ill and they need help for coming here. So most voluntary cases are difficult when you are giving them their medicine because they understand that they have to take it. (Participant 22; RMHN; Facility 1).

In contrast, some MHPs (e.g. psychiatrist and RMHN) suggested that consumers with little or no education, including those admitted involuntarily, participated less in the care plan. This notwithstanding, some MHPs said that irrespective of the background of consumers, they made several attempts to involve them as much as possible, as a fundamental human right to make them aware of their choices:

Having said all that as a fundamental human right I try to make sure that the patient is involved and is aware of the choices and makes the choices themselves to the best of their educational and you know socioeconomic background. (Participant 6; psychiatrist; Facility 3).

… we interview them to know why they are being admitted – whether it is voluntary or involuntary. Did they come by their own or they were forced. So if there were brought here involuntary then we explain to them why because some of the allegations are threatening. For example, there is an allegation that the consumer is threatening to kill someone and he's saying that he will commit suicide … That is involuntary admission. So we explain to them why they have come to this place and why there is the need to seek the services. (Participant 22; RMHN; Facility 1).

The analysis highlighted that therapeutic relationship is established through several mechanisms, according to the program theory. For example, some participants emphasised that the therapeutic relationship naturally starts by building rapport with the consumer and family caregivers; in particular, getting to know their condition while instilling hope and realistic expectations. Most participants found that the therapeutic relationship was realised through recognition of several ethical principles, which included respect for consumers’ privacy/confidentiality, dignity, preferences, comfort and seeking informed consent, as well as consumers’ backgrounds, including religion, gender, age, belief systems and culture. For example, some MHPs (e.g. RMHN) expressed that they ensured these ethical principles were upheld, to build the therapeutic relationship, to avoid being physically attacked or injured by angered consumers and to promote the quality of mental health services:

… every patient needs equal treatment, every patient has an equal right, you know with the condition they have a perception already, some hallucinate and some have a delusion, so if you discriminate and stigmatise and align with what the patient is thinking, it may cause the patient to become aggressive towards you the service provider. There is some time that we have a shortage of staff, so if the patient feels that you discriminate against him based on religious or cultural identity, they can attack you when you are alone in the ward. (Participant 26; psychiatrist; Facility 1).

A clinical psychologist with three years’ experience described that consumers having a Muslim background placed significance on the superiority of men over females. Therefore, MHPs could consider such cultural and religious perceptions when providing services to these consumers:

The participant described this issue as follows:

“ … a woman in a Muslim society does not have much recognition … Its cultural specifics, women are second to the man. So when a Muslim has a psychological condition and they walk into the consulting room and see a woman, their bias is certain. It depends on you the therapist to be competent enough to be able to overlook the fact that you are being looked down up based on your gender. That is very important, if not, you will not be able to give your best because you may already have a perception that he’s already looking down upon you. (Participant 27; Clinical Psychologist; Facility 1).

So when the client comes and he is not conversant with females you can approach your colleague to come and then talk to the client based on what the client believes in, so you are not here to undermine any religion, no we are to give our best of care so that is what we do. (Participant 13; RMHN; Facility 2).

As per the program theory, the contextual factors of evidence-based therapeutic process, together with the mechanisms, helped to achieve some positive effects on the outcomes of mental health services. For example, most MHPs expressed that the therapeutic relationship, as well as involvement in the consumer care plan, was relevant to promoting the quality of mental health services provided, particularly respecting consumers’ fundamental human rights and dignity, and helping consumers to be aware of their rights and choices. The MHPs said that the involvement of consumers and family caregivers helped to equip them to manage the condition, especially when discharged from psychiatric facilities. In particular, this involvement could enhance consumers’ knowledge and understanding as well as successful compliance with medications.

Further, three MHPs (clinical psychologist, occupational therapist, art therapist) also noted that the therapeutic relationship is very important to help consumers feel accepted, adhere to their prescribed medications, and involve them in the service:

Yes so therapeutic relationships start with rapport building, first getting to know the condition then you know how to approach the condition, so before you approach a patient you should know a little about what is going on, then you know the approach you are going to use in handling or engaging the patient and the family so you build rapport, let them be an integral part of the whole care you just don’t impose things on them because they also have a say in what is going on so that together we all can plan and give however it comes. (Participant 2; Occupational therapist; Facility 3).

Two MHPs further expressed that several recommendations had been made regarding the need to stop the wearing of uniforms as mandatory among providers, to avoid distinguishing consumers and providers on duty.

The MHPs expressed this as follows:

Some are even advocating that we shouldn’t wear uniform in nursing so that they will see that there is equality … you enter the ward and see that I am in mufti and you are also in mufti, you will see that these are the patient or the service providers … we are all equal. So when you come and we are doing recreational therapy, it is not just the patient who is dancing or singing … you realise that all the nurses are equally doing so. (Participant 26; RMHN; Facility 1).

Despite the increasing measures to build therapeutic alliances, the analysis demonstrated a contextual challenge related to barriers in consumer–provider communication, particularly for consumers with hearing impairments and those from linguistic minority backgrounds. Professional–consumer communication barriers were exacerbated by MPHs’ limited knowledge of sign language when communicating with consumers with hearing impairments. Most MHPs noted that while they did not know sign language, there was no designated sign language interpreter to support communication challenges for consumers with hearing impairments:

With people with disability, it is a difficulty that we are becoming more and more aware of … we do not necessarily have an insider translator for people who come and use sign language. (Participant 5; Psychiatrist; Facility 3).

No [no knowledge in sign language] so far we only have one patient that has a problem like that, that is how to sign but she even comes with her daughter who interprets for us so we don’t have to. (Participant 21; RMHN; Facility 1).

These people are naturally by their disabilities not able to access facilities like this. Especially when it comes to psychological services, being deaf and dumb and living in a society where the majority of the people do not accept such population makes it inaccessible. (Participant 27; Clinical Psychology; Facility 1)

Six MHPs narrated that, despite these challenges, they were able to use translators, mostly family caregivers and teachers or instructors, to provide sign language support. Participants occasionally relied on health professionals from other units who knew about basic signing to provide such support:

Sign language, for now, I think we have one person on the ward that understands sign language, so when the need arises we call that person to leave his/her unit and come and help here. (Participant 16; RMHN; Facility 2)

I am not trained in sign language I could do a few basic sign language but then not entirely everything but as I said I also don’t work alone I work with other professionals so if I would need to and get some kind of communication barrier or something then I have to rely on the other professionals. (Participant 8; Lay Art Therapist; Facility 3)

The analysis suggests that participants’ inability to provide adequate sign language interpretation for consumers with hearing impairments affected the quality of mental health services provided to such consumers. Also, an RMHN added that there are sometimes professional-consumer communication barriers, particularly for those who cannot communicate in the same language with the providers. The providers sometimes resort to help from other professionals who could communicate effectively in their language, as exemplified in the quote below:

Even sometimes language is a barrier … people come and depending on the staff they meet if they cannot speak their language, then it becomes a barrier. So most of the time, you have to find a colleague who can understand so that that person will do the interpretation. Even in the consulting room, we call someone to do the interpretation when the need arises. (Participant 26; RMHN; Facility 1)

## Discussion

This paper has answered the research question by identifying two context, mechanism and outcome (CMO) configurations that explain the evidence-based therapeutic process in mental health services. This CMO configuration includes that 1) technical competency stimulates evidence-based therapeutic process in mental health services, and 2) therapeutic alliance-building ensures effective interaction. The context-mechanism-outcome configurations identified from this analysis can facilitate the transferability of insights to other settings and context.

### CMO configuration two (theme one): technical competency stimulates evidence-based therapeutic process in mental health services

EBP in mental health services has been identified as fundamental to psychological growth and change [1, 2]. According to Donabedian middle-range theory on quality of care, the central tenet to evidence-based treatment is building the technical competency of MHPs [16]. Anthony, Ellison [2] have also concluded that the technical competency of MHPs could support consumers’ goal-setting and skills development. As a contextual factor in the current program theory, MHPs were perceived to have technical competencies, aligned with their specialist training, practical skills, certification, as well as with the regulatory bodies that monitored and supervised their services. The technical competency of MHPs was specifically achieved through continuous learning, in-service training and professional development planning, as well as being part of a multidisciplinary team. The program theory further suggests that these technical competencies could promote quality mental health services, particularly improved knowledge and skills on current treatment methods, ability to provide effective and efficient services, and enhancing consumers’ confidence and trust in the care. In particular, we identified that MHPs needed training in specific skills to improve their technical competence, in particular to provide holistic mental health services to consumers. For example, participants expressed the desire to gain skills in aggression and conflict management, psychological tools, customer care, health promotion, communication skills, infection prevention and dealing with difficult family caregivers. Although MHPs could obtain these skills as part of their professional training, refresher training in these areas is important as part of continuing efforts to provide holistic and integrated mental health services.

The findings of this study confirm previous studies in developed (eg. UK) [13, 17] and developing countries, which have recommended that in-service training and professional development plan are significant attributes for promoting the therapeutic relationship. For example Bee, Brooks [17] reported in the UK that the relational skills of professionals are the core facilitator of involving consumers in the care, though there appear some deficiencies in conventional staff training programs. More so, past studies have concluded that MHPs need to develop specific skills that promote the recovery journey of consumers [2, 16, 40, 41]. For example, Anthony, Ellison [2] recommended that MHPs providing mental health services and rehabilitation counselling be encouraged to gain skills in goal-setting as well as various therapies that could promote recovery of consumers. Such MHPs are encouraged to gain technical competencies in recovery based interventions, including integrated services (e.g. illness management, mindfulness-based interventions, music-creation therapy and active leisure or recreational activities), vocational rehabilitation [40, 42, 43], as well as narrative photovoice and art-making services [44–46]. Given this, the study recommends that government stakeholders should prioritise policy commitment and financial incentives that promote in-service skills development for MHPs, particularly in areas that promote evidence-based therapeutic processes.

### CMO configuration two (theme two): therapeutic relationship building ensures effective interaction

Contemporary care philosophies have widely advocated for consumer empowerment, contrary to the longstanding socio-medical constructs of mental health services [1, 2, 11]. Therapeutic relationship building constitutes measures used to strengthen such empowerment and involvement. Such a relationship could promote collaboration between MHPs and consumers and their family caregivers [1, 2, 47]. The study’s findings indicate that MHPs build therapeutic relationships with consumers and family caregivers through psychoeducation, consultation, diagnosis and family therapy sessions. These therapeutic relationships were used to involve consumers in care planning and in promoting adherence to ethical principles (e.g. respecting consumers’ privacy/confidentiality, dignity, preferences, informed consent and comfort, as well as, religious, gender, age and belief systems). Consistent with previous findings [4, 6, 15], the program theory demonstrated that the therapeutic alliance equipped consumers in illness management; for example, in adhering to medications. The therapeutic alliances were also relevant in promoting the personal recovery for consumers; thus, improving their knowledge and understanding, creating a sense of acceptance, hope and expectation, as well as active participation in mental health services. In similar findings from Canada, Thibeault [4] reported that a strategic approach such as being mindful was used by professionals to achieve therapeutic connection, and this helped consumers to demonstrate openness to engagement, willingness to share uncomfortable experiences, and visions of the future.

Therapeutic relationship building appears to be a relevant platform to promote the meaningful involvement of consumers in care planning. However, several factors could hinder or enable such therapeutic relationship building. Consistent with previous studies [3, 11, 12], our findings identified several individual as well as organisational factors that influenced therapeutic relationship building between consumers and MHPs. For example, individual factors such as consumers’ educational background affected the therapeutic relationship. In particular, consumers with little or no education as well as those from linguistic minority backgrounds were not adequately involved in the care plan, indicating a weak therapeutic relationship. Specifically, the findings confirm a previous study, which concludes that several consumer-related factors impeded the therapeutic relationship in psychiatric wards in southern Iran [3]. In previous findings, limited knowledge of consumers concerning their condition was a key factor that hindered effective therapeutic relationship with MHPs [3].

Moreover, the organisational factors that affected the building of a therapeutic relationship between consumers and MHPs were the philosophical approach to care and lack of sign language interpreters (nurse–consumer communication barrier). Consistent with previous findings [14, 17], the philosophical approach to practices, such as paternalistic and medical model traditions, which promote involuntary admission of consumers, was identified as a key hindrance to consumer participation in care planning, leading to a limited therapeutic relationship. For example, Bee, Brooks [17] concluded that while the technical skills of MHPs could promote consumer involvement, the philosophical tensions between consumers and professional accountability could impede therapeutic alliance-building in the UK secondary care mental health services.

Also, as per the program theory, contextual factors such as the lack of sign language interpreters in the mental health systems created barriers in provider–consumer communication, particularly for consumers with hearing impairments. The lack of sign language interpreters in providing communication support for consumers with hearing impairments could affect the quality of mental health services provided to them. This finding confirms previous studies, which have identified communication as a main contextual factor that could enable or hinder therapeutic relationship building [3, 6, 8, 12]. For example, Belcher and Jones [8] have suggested that provider–consumer communication involves a two-way process; thus listening to consumers problems, have a social conversation and further explaining to them any procedures to be undertaken. Providing adequate and effective verbal and non-verbal communication is an important part of the provider–consumer interaction. This position consumers to be equal partners and further help them to achieve wellness [6]. In addition, Haydon, van der Reit [12] have concluded that while provider–consumer communication is vital to promoting person-centred care, the use of humour in the therapeutic relationship should not go unrecognised. Some studies have also recommended that MHPs should consider humour as a useful tool in the therapeutic relationship [6, 13]. Given this, stakeholders are encouraged to enforce and monitor policies and regulations that support adequate provision of sign language interpreters in all levels of mental health service provision.

## Limitations

Several limitations need to be acknowledged. First, the study was limited to responses from purposively selected MHPs in three psychiatric facilities, without the perspectives of mental health policy planners from government ministries or from family caregivers. Despite this, the researchers have enhanced the trustworthiness of data collection and documentation according to the seven criteria of Pawson and Tilley [39], which are transparency, accuracy, purposivity, utility, propriety, accessibility and specificity. The methodology informing this context, mechanism and outcome configuration was collaboratively developed by experts in mixed-methods research. The research team first developed, confirmed and discussed the methodology before implementing it. Moreover, the thematic analysis process was subjected to coding by consensus, member checking and a series of debriefing sessions. The findings have been compared with those of the international literature on quality mental health services.

## Conclusion

This study sought to use a realistic evaluation to explore the evidence-based therapeutic process in mental health services, from the perspective of MHPs. As per the program theory, contextual factors such as the technical competencies of MHPs stimulated the evidence-based therapeutic process in mental health services. For example, MHPs perceived that they had technical competencies, attained through mechanisms such as continuous learning, in-service training, professional development planning and the multidisciplinary team environment, as well as through clinical and academic training sessions. As per the program theory, these contextual factors and mechanisms were relevant to achieving outcomes such as mental health service quality through reduced hospitalisation days, improved knowledge and skills in MHPs on current treatment methods, as well as building the confidence level of consumers. Contextually, the MHPs established therapeutic relationship through psychoeducation, consultation, diagnosis and family therapy sessions. These therapeutic alliance were built through mechanisms such as involvement of consumers in the care plan, considering consumers’ individual factors (e.g. education background), philosophy of mental health services (e.g. voluntarily or involuntarily nature of admission), building rapport and adhering to high ethical principles (e.g. dignity and respect, choices, respecting consumers privacy/confidentiality, dignity, preferences, informed consent, comfort as well as religious, gender, age and belief systems). As per the program theory, these contextual factors and mechanisms helped to equip consumers in management of their conditions; enhanced consumers’ knowledge and understanding, compliance with medications, active participation in the care plan, feeling accepted, having hope and expectations, and making the service participatory. Despite these factors, there was a barrier to consumer–provider communication, particularly for consumers with hearing impairments and those from linguistic minority backgrounds. This contextual challenge resulted in communication barriers for this category of consumers.

### Implications for mental health policy and practice

The study’s findings have identified some contextual factors and mechanisms that could enhance the evidence-based therapeutic process in mental health service delivery. The findings are relevant to informing policy, mental health nursing practices and the education of MHPs and students. As per the program theory, we recommend that government stakeholders and policymakers should prioritise the current mechanisms used to promote technical competencies for MHPs. For example, policies, periodic monitoring and adequate financial incentives should be provided to support continuing in-service training as well as professional development planning for MHPs. Health service planners and administrators are encouraged to prioritise ongoing clinical and academic training sessions as well as collaborative and multidisciplinary teamwork. Such measures could enhance the technical competencies of mental health professionals and subsequently improve the therapeutic process in the services. As per the program theory, the current mechanisms used to promote therapeutic relationships between mental health professionals and consumers and their family caregivers should be promoted and monitored periodically to ensure effective consumer involvement and participation in the care plan. In particular, mental health professionals should be encouraged to adhere to the existing ethical principles as well as promote a consumer-centred or strength-based approach to therapeutic relationship building. These principles should be integrated into the routine practice to ensure that consumers are involved at all stages of the service delivery. Such integration could help mental health professionals to adhere to treatment guidelines. These mechanisms could also help consumers to adhere to treatment and medication, and further promote their clinical and personal recovery journey.

Moreover, based on the contextual challenges – for example, consumer–provider communication barriers – government stakeholders and health services planners are encouraged to prioritise legislation that supports the provision of sign language interpreters to support consumers with hearing impairments. Finally, the identified program theory enhancing the evidence-based therapeutic process should be prioritised in educational programs for mental health professionals and students.

## Supplementary Information

**Additional file 1.**

## Data Availability

The datasets used and/or analysed during the current study are available from the corresponding author on reasonable request. All data collection tools, including the interview guide, have been uploaded as supplementary files.

## Abbreviations

CMHN: Community Mental Health Nursing
CMHO: Community Mental Health Office
CMO: Context + Mechanism = Outcome
CPDs: Continuing Professional Development
ECT: Electroconvulsive therapy
EBP: Evidenced-based practises
HREC: Human Research Ethics Committee
MHPs: Mental Health Professionals
RMN: Registered Mental Health Nursing

## References

[1] Anthony WA, Mizock L (2014). Evidence-based processes in an era of recovery: implications for rehabilitation counseling and research. Rehabil Counsel Bull.

[2] Anthony WA, Ellison ML, Rogers ES, Mizock L, Lyass A (2014). Implementing and evaluating goal setting in a statewide psychiatric rehabilitation program. Rehabil Counsel Bull.

[3] Pazargadi M, Fereidooni Moghadam M, Fallahi Khoshknab M, Alijani Renani H, Molazem Z (2015). The therapeutic relationship in the shadow: nurses’ experiences of barriers to the nurse–patient relationship in the psychiatric ward. Issues Ment Health Nurs.

[4] Thibeault C (2016). An interpretation of nurse–patient relationships in inpatient psychiatry: understanding the mindful approach. Global Qual Nurs Res.

[5] Kornhaber R, Walsh K, Duff J, Walker K (2016). Enhancing adult therapeutic interpersonal relationships in the acute health care setting: an integrative review. J Multidiscip Healthc.

[6] Pullen RL, Mathias T (2010). Fostering therapeutic nurse-patient relationships.

[7] Dziopa F, Ahern K (2009). Three different ways mental health nurses develop quality therapeutic relationships. Issues Ment Health Nurs.

[8] Belcher M, Jones LK (2009). Graduate nurses’ experiences of developing trust in the nurse–patient relationship. Contemp Nurse.

[9] Macdonald LM (2016). Expertise in everyday nurse–patient conversations: the importance of small talk. Global Qual Nurs Res.

[10] Dziopa F, Ahern KJ (2009). What makes a quality therapeutic relationship in psychiatric/mental health nursing: A review of the research literature. Internet J Adv Nurs Pract.

[11] Harris BA, Panozzo G (2019). Therapeutic alliance, relationship building, and communication strategies-for the schizophrenia population: an integrative review. Arch Psychiatr Nurs.

[12] Haydon G, van der Reit P, Browne G (2015). A narrative inquiry: humour and gender differences in the therapeutic relationship between nurses and their patients. Contemp Nurse.

[13] Majumder P, Vostanis P, Karim K, Oreilly M. Potential barriers in the therapeutic relationship in unaccompanied refugee minors in mental health. J Ment Health. 2018;28(4):372–8. 10.1080/09638237.2018.1466045 29688140

[14] Harris B, Panozzo G (2019). Barriers to recovery-focused care within therapeutic relationships in nursing: attitudes and perceptions. Int J Ment Health Nurs.

[15] Hartley S, Raphael J, Lovell K, Berry K (2020). Effective nurse–patient relationships in mental health care: a systematic review of interventions to improve the therapeutic alliance. Int J Nurs Stud.

[16] Badu E, O’Brien AP, Mitchell R (2019). The conceptualization of mental health service quality assessment: consumer perspective. Adm Policy Ment Health Ment Health Serv Res.

[17] Bee P, Brooks H, Fraser C, Lovell K (2015). Professional perspectives on service user and carer involvement in mental health care planning: a qualitative study. Int J Nurs Stud.

[18] Alshammari M, Duff J, Guilhermino M (2019). Barriers to nurse–patient communication in Saudi Arabia: an integrative review. BMC Nurs.

[19] Badu E, O’Brien AP, Mitchell R (2018). An integrative review of potential enablers and barriers to accessing mental health services in Ghana. Health Res Policy Syst.

[20] Deborah TD, Badu E, Amy BA, Josephine AN, Gyamfi N, Opoku MP. The burden of caregiving among mental health nurses providing services to consumers with depression in Ghana. Perspect Psychiatric Care. 2019. 10.1111/ppc.12377 30920680

[21] Badu E, Mitchell R, O’Brien AP (2019). Pathways to mental health treatment in Ghana: challenging biomedical methods from herbal-and faith-healing perspectives. Int J Soc Psychiatry.

[22] Nartey AK, Badu E, Agyei-Baffour P, Gyamfi N, Opoku MP, O'Brien AP, Mitchell R (2019). The predictors of treatment pathways to mental health services among consumers in Ghana. Perspect Psychiatric Care.

[23] D'Antonio P, Beeber L, Sills G, Naegle M (2014). The future in the past: H ildegard P eplau and interpersonal relations in nursing. Nurs Inq.

[24] Peplau HE (1997). Peplau's theory of interpersonal relations. Nurs Sci Q.

[25] Senn JF (2013). Peplau’s theory of interpersonal relations: application in emergency and rural nursing. Nurs Sci Q.

[26] Badu E, O’Brien AP, Mitchell R (2019). An integrative review on methodological considerations in mental health research–design, sampling, data collection procedure and quality assurance. Arch Public Health.

[27] Astbury BGP (2011). The practice of realistic evaluation: a conceptual and empirical review.

[28] Mutschler C, Rouse J, McShane K, Habal-Brosek C (2018). Developing a realist theory of psychosocial rehabilitation: the clubhouse model. BMC Health Serv Res.

[29] Badu E, O’Brien AP, Mitchell R, Osei A (2020). Mediation and moderation effects of health system structure and process on the quality of mental health services in Ghana–structural equation modelling. PLoS One.

[30] Badu E, O'Brien AP, Mitchell R, Osei A. Factors associated with the quality of mental health services and consumers' functionality using tertiary-based services. Perspect Psychiatric Care. 2021. 10.1111/ppc.12820. 10.1111/ppc.12820 33942311

[31] Adams A, Sedalia S, McNab S, Sarker M (2016). Lessons learned in using realist evaluation to assess maternal and newborn health programming in rural Bangladesh. Health Policy Plan.

[32] Jagosh J, Bush PL, Salsberg J, Macaulay AC, Greenhalgh T, Wong G, Cargo M, Green LW, Herbert CP, Pluye P (2015). A realist evaluation of community-based participatory research: partnership synergy, trust building and related ripple effects. BMC Public Health.

[33] Flynn R, Rotter T, Hartfield D, Newton AS, Scott SD (2019). A realist evaluation to identify contexts and mechanisms that enabled and hindered implementation and had an effect on sustainability of a lean intervention in pediatric healthcare. BMC Health Serv Res.

[34] Duncan C, Weich S, Fenton S-J, Twigg L, Moon G, Madan J, Singh SP, Crepaz-Keay D, Parsons H, Bhui K (2018). A realist approach to the evaluation of complex mental health interventions. Br J Psychiatry.

[35] Stokes T, Atmore C, Penno E, Richard L, Wyeth E, Richards R, Doolan-Noble F, Gray AR, Sullivan T, Gauld R (2019). Protocol for a mixed methods realist evaluation of regional district health board groupings in New Zealand. BMJ Open.

[36] Bertotti M, Frostick C, Hutt P, Sohanpal R, Carnes D (2018). A realist evaluation of social prescribing: an exploration into the context and mechanisms underpinning a pathway linking primary care with the voluntary sector. Primary Health Care Res Dev.

[37] Braun V, Clarke V (2006). Using thematic analysis in psychology. Qual Res Psychol.

[38] Saldaña J (2015). The coding manual for qualitative researchers.

[39] Pawson R, Tilley N. An introduction to scientific realist evaluation. Evaluation for the 21st century: A handbook. 1997;1997:405–18.

[40] Gyamfi N, Badu E, Mprah WK, Mensah I (2020). Recovery services and expectation of consumers and mental health professionals in community-based residential facilities of Ghana. BMC Psychiatry.

[41] Gyamfi N, Bhullar N, Islam MS, Usher K (2020). Knowledge and attitudes of mental health professionals and students regarding recovery: a systematic review. Int J Ment Health Nurs.

[42] Brooke-Sumner C, Lund C, Selohilwe O, Petersen I (2017). Community-based psychosocial rehabilitation for schizophrenia service users in the north west province of South Africa: a formative study. Soc Work Ment Health.

[43] Salyers MP, McGuire AB, Kukla M, Fukui S, Lysaker PH, Mueser KT (2014). A randomized controlled trial of illness management and recovery with an active control group. Psychiatr Serv.

[44] Mizock L, Russinova Z, DeCastro S (2015). Recovery narrative Photovoice: feasibility of a writing and photography intervention for serious mental illnesses. Psychiatric Rehabil Jl.

[45] Mizock L, Russinova Z, Shani R (2014). New roads paved on losses: Photovoice perspectives about recovery from mental illness. Qual Health Res.

[46] Clements K (2012). Participatory action research and photovoice in a psychiatric nursing/clubhouse collaboration exploring recovery narrative. J Psychiatr Ment Health Nurs.

[47] Schroder A, Larsson BW, Ahlstrom G (2007). Quality in psychiatric care: an instrument evaluating patients' expectations and experiences. Int J Health Care Qual Assurance.

