# Supplementary material for: A qualitative study of evidence-based therapeutic process in mental health services in Ghana– context-mechanisms-outcomes
Source: BMC Health Serv Res. 2021 Sep 25;21:1013. doi: 10.1186/s12913-021-06993-1 (PMC8466714; doi:10.1186/s12913-021-06993-1)
Supplement: Supplementary file 1 — Additional file 1. [file 12913_2021_6993_MOESM1_ESM.doc]

**Interview guide – Mental Health Professionals**

Professor Anthony Paul O’Brien

School of Nursing and Midwifery, School of Public Health and Medicine

The University of Newcastle (UON), University Drive, Callaghan NSW 2308 Australia

Tel: +61 2 49854368 Mob: +61 (0)488941943

[tony.obrien@newcastle.edu.au](mailto:tony.obrien@newcastle.edu.au)

**Interview guide for the Research Project:**

**The quality of mental health services in Ghana: Providers and Consumers perspective**

*Dear Sir/Madam,*

You are invited to participate in the research project identified above, which is being conducted by PhD student - Eric Badu. Eric is enrolled at School of Nursing and Midwifery, Faculty of Health and Medicine at the University of Newcastle, NSW, Australia. Eric is supervised by Professor Anthony Paul O’Brien and Professor Rebecca Mitchell.

*The attached interview guide asks you to respond to questions concerning your experiences with the quality of mental health services you provide to consumers. The interview is part of my doctoral studies being supervised by the University of Newcastle. It is appreciated if you could participate in this interview. Your opinions are highly valued and essential to improve the kind of mental health service in Ghana****.*** *Your response will be recorded using an audio tape recorder, however, your name or any other personal information will NOT be used in the research study to maintain your confidentiality and anonymity. No individual will be identified accidentally. No individual details will be disclosed to anyone. Your interview will be transcribed using a false name (pseudonym). Code numbers and pseudonyms will be used in place of names throughout the research process.*

***Questions***

1. **Tell me about the technical competency of mental health professionals in this facility?**

Probe

1. What is the technical competency of providers? Clues –clinicians’ knowledge about appropriate intervention, ability to use best practices and to accurately assess consumer problems?
2. How does this work in practice?
3. How does this contribute to the quality of mental health services?
4. **Tell me about the training and professional development plan and programmes available for you?**

Probe

1. Have you attended any in-service training this year?
2. How often do you attend the in-service training? In which areas do you receive in-service training?
3. What are the training needs?
4. How does this contribute to the quality of mental health services?
5. **Tell me about the extent of access to mental health services to consumers?**

Probe

1. How do you ensure equity of access regardless of gender, disability status, age group and geographical location? Clues – considering the needs of people with language issues, information for consumers and family caregivers, medical records?
2. How does this contribute to the quality of mental health services?
3. **Tell me about the extent at which consumers are involved in the treatment plans?**

Probe

1. How do you involve consumers in the formulation and delivery of their care? *Clue – the treatment plans are individually tailored for each consumer? Consumers are informed, where appropriate, about the reasons for referral to a specialist or other professionals? listening to consumers and taking them seriously? Mental health professionals are aware of the potential impact of a mental health problem on consumers’ behaviour? Mental health professionals are aware of stigmatizing attitudes against consumers?*
2. How do you handle the therapeutic relationship with consumers? *Clues Treating consumers with respect, courtesy and considerations irrespective of age, gender, religion and cultural identity?*
3. How does this contribute to quality mental health services?
4. **Tell me about your experiences working with consumers of mental health services?**

Probe

1. How does their socio-demographic background facilitate the quality of mental health services? Clues – the c*linical history, gender, cultural issues?*
2. How does your therapeutic relationship with consumers influence the quality of mental health services?
